# Biological Implications in Cassava for the Production of Amylose-Free Starch: Impact on Root Yield and Related Traits

**DOI:** 10.3389/fpls.2016.00604

**Published:** 2016-05-20

**Authors:** Amanda Karlström, Fernando Calle, Sandra Salazar, Nelson Morante, Dominique Dufour, Hernán Ceballos

**Affiliations:** ^1^Department of Plant Breeding, Swedish University of Agricultural Sciences, AlnarpSweden; ^2^Centro Internacional de Agricultura Tropical, PalmiraColombia; ^3^Centre de Coopération Internationale en Recherche Agronomique pour le Développement, UMR Qualisud, MontpellierFrance

**Keywords:** waxy starch, yield penalty, cassava markets, economic impact, root and tuber crops

## Abstract

Cassava (*Manihot esculenta*, Crantz) is an important food security crop, but it is becoming an important raw material for different industrial applications. Cassava is the second most important source of starch worldwide. Novel starch properties are of interest to the starch industry, and one them is the recently identified amylose-free (waxy) cassava starch. Waxy mutants have been found in different crops and have been often associated with a yield penalty. There are ongoing efforts to develop commercial cassava varieties with amylose-free starch. However, little information is available regarding the biological and agronomic implications of starch mutations in cassava, nor in other root and tuber crops. In this study, siblings from eight full-sib families, segregating for the waxy trait, were used to determine if the mutation has implications for yield, dry matter content (DMC) and harvest index in cassava. A total of 87 waxy and 87 wild-type starch genotypes from the eight families were used in the study. The only significant effect of starch type was on DMC (*p* < 0.01), with waxy clones having a 0.8% lower content than their wild type counterparts. There was no effect of starch type on fresh root yield (FRY), adjusted FRY and harvest index. It is not clear if lower DMC is a pleiotropic effect of the waxy starch mutation or else the result of linked genes introgressed along with the mutation. It is expected that commercial waxy cassava varieties will have competitive FRYs but special efforts will be required to attain adequate DMCs. This study contributes to the limited knowledge available of the impact of starch mutations on the agronomic performance of root and tuber crops.

## Introduction

Cassava (*Manihot esculenta*, Crantz) is an important source of food calories in sub-Saharan Africa, fulfilling a critical role as a food security crop (Haggblade et al., 2012). Its roots are also one of the most important sources of commercial starch in tropical and subtropical countries (Moorthy, 2004). In fact, the crop is the second most important source of starch worldwide, after maize, and the most traded one (Stapleton, 2012). The global export of cassava starch and flour in 2014 amounted to 8.5 million tons (Food and Agriculture Organization [FAO], 2015). In South and South-East Asia, starch export has been one of the drivers of cassava expansion and 40% of the total cassava production is used for starch extraction (Onwueme, 2002; Fuglie et al., 2006). Applications of cassava starch (also known as tapioca) is found in the textile and pharmaceutical industry and within food manufacturing, for which it is well suited since it has a bland taste and produces a clear paste (Jobling, 2004; Fuglie et al., 2006; Food and Agriculture Organization [FAO], 2015).

Amylose-free (or waxy) starch phenotype was first identified in maize germplasm from China (Collins, 1909; Collins and Kempton, 1914) and few years later in the segregating progeny from a landrace (Sanford’s White Flint) in Connecticut (Mangelsdorf, 1924). The recessive nature of this trait was also reported about a century ago (Collins and Kempton, 1914; Mangelsdorf, 1924; Kiesselbach and Petersen, 1926). During the Second World War the United States could not import tapioca from Thailand. The industry searched for alternatives to the cassava starch it could no longer import and found that functional properties of waxy maize starch resembled more closely (than wild type maize) those of tapioca. The industry of waxy maize was thus borne (Fergason, 2001). Spontaneous waxy starch mutations have been found in barley, wheat, rice, potato and sorghum (Wang et al., 1995; Graybosch, 1998; McPherson and Jane, 1999; Song and Jane, 2000). Low amylose genotypes of yam have also been identified (Pérez et al., 2011). Compared to their wild-type counterparts, amylose-free starches generally show a higher swelling power, higher peak viscosity, clearer pastes and a better freeze-thaw stability, which are highly desirable properties in many food applications (Jobling, 2004).

In spite of the relevance of cassava for the starch industry, no commercial cassava varieties offering special functional properties has ever been released. Amylose content has profound effect on starch functional properties in different crops as stated above. The average starch content of the cassava accessions in the FAO cassava germplasm collection is 84.5% (on a dry root basis) and the average amylose content is 20.7%, ranging between 15.2 and 26.5% (Sánchez et al., 2009). It was only in 2006 that a cassava mutant lacking amylose in the starch was discovered at the International Center for Tropical Agriculture (CIAT). The discovery came as result of self-pollinations of a large number of cassava genotypes, conducted to bring forward low-frequency recessive traits in the crop (Ceballos et al., 2007). Recently new sources of amylose-free cassava have been identified (Morante et al., 2016).The development of varieties with waxy starch had long been an objective for the cassava community. It was envisioned that waxy varieties could better fit the needs for specific uses and, therefore, entail higher selling prices of the roots and/or strengthen markets for cassava. In other words, a commercial variety with amylose-free starch would benefit both, farmers and processors. The waxy starch trait is so important that successful transgenic approaches to down-regulate *GBSSI* have also been conducted (Raemakers et al., 2003, 2005; Zhao et al., 2011).

The lack of amylose in waxy genotypes has been shown to be the result of a recessive mutation in the *wx* locus encoding granule-bound starch synthase I (GBSSI), the protein responsible for the elongation of amylose in the starch granule (Fukunaga et al., 2002; Larkin and Park, 2003; Ceballos et al., 2007; McIntyre et al., 2008). The full-length sequence of the cassava *GBSSI* gene has been determined (Aiemnaka et al., 2012) and single-nucleotide amplified polymorphism (SNAP) markers has been developed to differentiate waxy (*wx wx*) from non-waxy heterozygous (*Wx wx*) and non-waxy homozygous (*Wx Wx*) genotypes. In most cases waxy starches lack completely amylose. However, in some cases small amount of amylose (<5%) can be found in certain waxy mutations in maize (Andres and Basciollo, cited by Fergason, 2001). In the case of cassava spontaneous mutations in cassava (Ceballos et al., 2007; Morante et al., 2016) have no amylose, whereas transgenic lines vary from about 2–10% of amylose (Zhao et al., 2011; Koehorst-van Putten et al., 2012).

Since the discovery of the waxy starch mutation in cassava several studies have been conducted to characterize its functional properties (Ceballos et al., 2007; Sánchez et al., 2010; Rolland-Sabaté et al., 2012, 2013). Compared to other waxy and non-waxy starches, waxy cassava starch has been shown to have improved freeze-thaw stability (Raemakers et al., 2005; Sánchez et al., 2010; Dufour et al., 2013) as also reported in potato (Jobling et al., 2002). In addition, waxy cassava starch was reported to have a clearer paste and a higher peak viscosity than other waxy and non-waxy starches, with the exception of potato (Sánchez et al., 2010). The starch content of the first non-transgenic waxy cassava genotype (AM 206-5) was shown to be comparable to that of two wild-type starch varieties (Ceballos et al., 2007).

The advantages of the *in planta* versus *in vitro* modification of starch functional properties has been already reported (Slattery et al., 2000; Davis et al., 2003). Waxy starches from root and tuber crops (e.g., cassava and potato) offer the advantage of clearer gels, bland or neutral flavor and taste (Koehorst-van Putten et al., 2012) and higher viscosities and different gel textures (Sánchez et al., 2010) compared with those from cereals. It is undeniable that waxy cassava starch offers enough advantages for the industry. Consequently, projects to develop non-transgenic commercial cassava varieties producing amylose-free starch have been initiated (Aiemnaka et al., 2012) with the support of the private sector. This is the first time ever that the starch industry invests in cassava breeding and it is an evidence of the interest generated by this waxy starch. However, it is not clear what the biological impact is when a cassava plant produces amylose-free starch. For a commercial variety and the entire value chain to be successful, the advantages derived from the special properties of the waxy starch must not be counterbalanced by potential agronomic disadvantages of the varieties producing it. The objective of this study, therefore, was to determine the effect on cassava yield and yield components of the waxy trait in cassava (Karlström, 2015).

## Materials and Methods

### Parental Material and Population Development

This study took advantage of eight full sib families (same male and female progenitor) segregating for the mutation at the *GBSSI* locus. The families were developed from F_1_ plants which were obtained from crosses between the first identified source of waxy starch (AM 206-5) and eight elite varieties with good agronomic characteristics adapted to the main cassava growing environments in Colombia (except highlands). The F_1_ plants (heterozygous for the waxy allele) from unrelated families were crossed to produce a pseudo-F_2_ generation. **Table 1** describes the full-sib families from this F_2_ generation. Starch type was identified by screening the roots with the reliable iodine staining technique first reported by Weatherwax (1922). Waxy roots are typically stained reddish-brown by the iodine solution, whereas wild type starch roots stain dark-blue. Homozygote (*Wx Wx*) and heterozygote (*Wx wx*) wild type plants were not distinguished in the study.

**Table 1:** Description of family size, number of selected plants and distribution within sets for each of the eight families.

Family	Selected (waxy + wild type)	Set 1	Set 2	Set 3
GM 5458	10+10	3	3	4
GM 5466	15+15	5	5	5
GM 5507	8+8	3	3	2
GM 5536	8+8	3	3	2
GM 5615	8+8	3	3	2
GM 5619	8+8	3	3	2
GM 5672	15+15	5	5	5
GM 5722	15+15	5	5	5
**Total**	**87+87**	**30**	**30**	**27**

**FIGURE 1:**
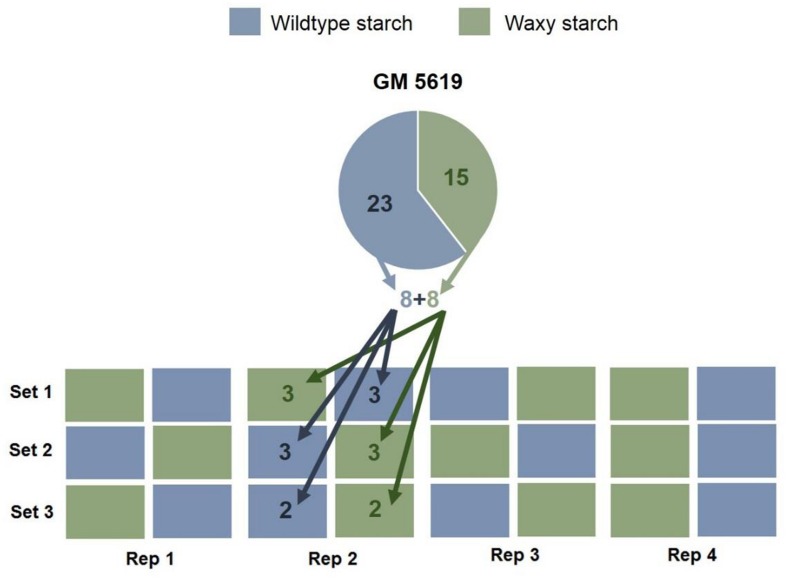
**Illustration of how the genotypes from each of the eight families were used to obtain random and representative samples.** Family GM 5619 had 23 and 15 genotypes producing wildtype and waxy starch, respectively. Eight waxy and eight wild-type genotypes were randomly chosen and allocated into three sets (3+3+2 = 8 genotypes). Each set was then replicated four times. The waxy and wildtype starch genotypes from the other seven families were similarly distributed for a total of 30 plants in each plot.

The number of genotypes in each family varied and, therefore, each family was represented by a different number of genotypes (**Table 1**). However, for each family, an equal number of waxy and wild type genotypes were chosen (87 genotypes per type of starch). As seen in **Figure 1**, family GM 5619 (used as an example) had 38 genotypes, with 15 of them producing waxy starch and the remaining 23 with amylose-containing starch. A total of eight genotypes among the group of the 15 producing waxy starch were randomly chosen. Similarly, eight genotypes with wild type starch out of the 23 found in this family were randomly picked. It is assumed that each of the two groups of eight genotypes are random samples of the genetic diversity of this family, with the exception that one group produced waxy starch and the other group produced wild type starch. A similar approach was taken for the seven remaining families.

### Field Evaluation

The effects of the waxy trait on yield and yield components were evaluated based on data from a field-trial conducted at CIAT experimental station, Palmira, Valle del Cauca, Colombia. Soils at this station are fine-silty, mixed, isohyperthermic Aquic Hapludol (Duque-Vargas et al., 1994). Standard cultural practices were used for this evaluation, providing irrigation when necessary, and keeping the main pest problems (particularly the whitefly *Aleurotrachellus socialis* and different mite species) under control.

### Experimental Design

A special field design was used for a truly randomized distribution of the plants and genotypes involved in the study. Instead of planting all genotypes from a certain family together, they were more or less equally split into three different sets (**Table 1**). Each set had a combination of about 1/3 of the genotypes from each of the eight families and were all planted simultaneously in the same field. Each set contained a total of 30 waxy and 30 wild type genotypes from the same F_2_ families except set 3 which only had 27 genotypes of each starch type (**Figure 1**). Therefore, three additional genotypes were added to the third set as filling (they were not included in the analysis of results). The three sets of genotypes were replicated in four blocks. In other words, the same genotype was cloned and replicated four times (one plant per genotype and block). This added up to a total of 348 waxy and 348 wildtype plants (87 × 4 = 348). In each plot with 30 plants, planting was done in five rows with six plants per row. The plants were harvested, as usual by hand, at 11 months after planting.

The data collected at harvest were fresh root yield (FRY), harvest index (HIN, root weight/total weight of biomass including roots) and dry matter content (DMC). HIN is usually used as an indirect yield indicator in cassava after reports of a higher correlation between HIN in single row trials and FRY performance in replicated plots in multi-location trials than the correlation between root yields in the same type of trials (Kawano et al., 1978). Because of the volume and weight of a total of 30 plants at harvest, measurements were done on a total per row within each plot. Results from the six rows in each plot were then combined for further analysis. Data from individual genotypes, therefore, were not available. FRY was measured by bulking the harvested roots from the five plants of each row and then adding the weights across the six rows from each plot. The FRY per hectare (t ha^-1^) was then estimated taking into account the area of each plot. Roots with symptoms of rotting were discarded and not included in the measurement. When plants were missing an adjustment for missing plants was used in the estimation of FRY (Ad.FRY; Pérez et al., 2010). HIN was also estimated by bulking the weight of roots and above ground biomass from the plants of each row. Averages for each plot were then calculated. DMC was estimated using the gravimetric method (Kawano et al., 1978; Toro and Cañas, 2012) by bulking the roots from each row, with two replicated measurements per row. The two measurements per row were then used to calculate the average DMC across the six rows in each plot. Therefore a total of 12 independent estimations for DMC among the 30 plants in each plot were used for the average DMC.

### Statistical Analysis

The main question to answer by this study is the biological impact of the waxy starch mutation in key agronomic traits. Although there is genetic variation among the 87 genotypes (within each type of starch), analysis was done on the bulked data. Individual genotypes within each starch group were randomly chosen. Genotypes representing each group are considered to be random samples of the genetic variation within each family. The only difference being the production of waxy or amylose-containing starch. Although variation among individual genotypes is relevant, the main objective of this study focused on the impact of the mutation across different genotypes. The data for each plot (bulked across rows) was used for the analysis of variance. Because of the field design the statistical analysis were conducted with the sets nested within replicate effects and starch type nested within set effects. All sources of variation in the analysis of variance were considered random, except for the type of starch. Statistical analysis was made using the SAS Software and the Proc GLM procedure (SAS, 2008).

## Results

**FIGURE 2:**
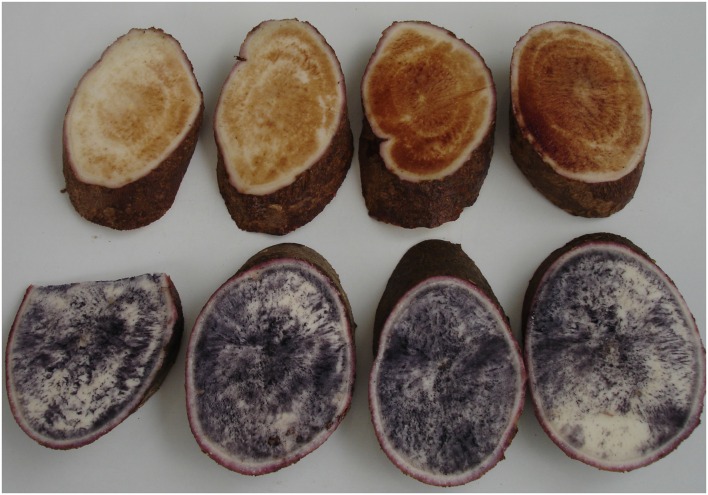
**Illustration of the staining technique based on an iodine solution used to identify roots with waxy starch (staining reddish) or amylose-containing wild type starch (staining blue)**.

Plant germination and growth was satisfactory. Environmental conditions were drier than normal. Since irrigation was available when necessary, plants did not suffer drought stress but grew under slightly higher than normal average temperatures and lower relative moisture in the air. Averages for FRY, HIN and DMC of the waxy genotypes and their wild type sibling counterparts are presented in **Table 2**. Starch type was confirmed using the efficient and reliable iodine test (**Figure 2**).

**Table 2:** Means and standard deviations of FRY, adjusted FRY, DMC, and HI for the waxy and wild type starch cassava full-siblings.

Starch type	FRY (t ha^-1^)	Ad.FRY (t ha^-1^)	DMC (%)	HIN (0–1)
Waxy	7.24 (± 2.40)	7.57 (± 2.58)	32.8 (± 1.49)	0.41 (± 0.08)
Wild-type	7.40 (± 2.73)	7.67 (± 3.61)	33.6 (± 1.61)	0.38 (± 0.07)
LSD_0.05_	0.91	0.94	0.46	0.02
LSD_0.01_	1.21	1.25	0.61	0.03

*Also included is the least significant difference (LSD) at the 0.05 and 0.01 level for the analysis of variance.*

Starch type (waxy versus wild type) had no significant effect on FRY, with similar results when yields were adjusted to take missing plants into consideration. Only few plants were missing in the entire experiment and, therefore, an agreement between the two ways to assess FRY was expected. The only significant effect on FRY and Ad.FRY was for the replication source of variation (*p* ≤ 0.05). Likewise, there was no significant effect of starch type on HIN, while both replication and set within replications had highly significant (*p* ≤ 0.01) effects (**Table 3**).

**Table 3:** Mean squares from the analysis of variance for FRY, adjusted FRY, DMC, and HI in eight full sibling families segregating for the waxy trait.

Source of variation	df	FRY	Ad.FRY	DMC	HIN
Replicate	3	24.4ˆ*	26.8ˆ*	7.8ˆ**	0.056ˆ**
Set (Replicate)	8	9.2	10.3	6.2ˆ**	0.013ˆ**
Starch type (Set* Replicate)	12	2.9	3.7	6.1ˆ**	0.005
Error	96	6.3	6.7	1.6	0.004
Coefficient of variation	-	34.2	34.1	3.8	16.4

*,**Significant at the 0.05 and 0.01 probability level, respectively.

There was a highly significant effect (*p* ≤ 0.01), on the other hand, of starch type on DMC. In fact this is the only case where starch type had a significant effect in any variable. Average DMC in waxy starch genotypes was 32.8%, whereas in wild type starch genotypes the average was 33.6%. The difference of 0.8% is small, but nonetheless, highly significant from a statistical point of view. Replication and sets within replication also showed highly significant (*p* ≤ 0.01) effects on DMC (**Tables 2** and **3**).

## Discussion

The present evaluation aims at assessing the biological implication for cassava (without any previous selection) to produce roots with waxy starch. The average performance of the waxy clones compared with their wild type counterparts is useful only for understanding the biological impact of the mutation. Results from this study suggest that selected clones with waxy starch will be competitive regarding FRY. Results for DMC, on the other hand, raised some concerns since predictions indicate lower DMC on waxy genotypes. Data from individual genotypes was not taken because the main interest focused on the differences in the average performance of waxy versus wild-type genotypes. Therefore, results from this study are not able to determine if individual clone(s) producing amylose-free starch can or cannot reach competitive levels of DMC as well. This distinction is important because it is outstanding individual (waxy) cassava clones which will be grown commercially by farmers, not the entire full sib families, but the study was not designed to address this issue.

The most advanced program to develop waxy commercial cassava varieties (in Thailand) faced problems selecting progenies with DMC comparable with those of commercial varieties, whereas for FRY results were comparable for both types of starch (Rodjanaridpiched et al., 2012). Preliminary results from the ongoing work to develop amylose-free commercial cassava varieties in Colombia also points to difficulties in achieving the levels of DMC observed in commercial checks (data not presented). However, differences in DMC comparing commercial checks and selected waxy starch clones were as large as 3–4% (Rodjanaridpiched et al., 2012). The present study, on the other hand, reports differences in DMC of less than 1%. Although, the penalty on DMC is relatively small (**Table 2**), it could potentially become a quantitatively important economic loss in large scale processing of (waxy) cassava starch. However, yield penalty (through a reduced DMC) should not be as large as those reported in the first batch of waxy cassava clones reported by Rodjanaridpiched and co-workers in 2012. Results from this study suggest that the next selection cycle of breeding for waxy cassava starch should focus particularly in identifying materials with higher DMC.

Results from Thailand and Colombia compared preliminary selections of waxy cassava varieties (out of less than 3000 initial genotypes) with the best commercial varieties available (which had been selected after many years of evaluation and arose after testing 10s of 1000s of genotypes). They are, therefore unfair comparisons which would tend to overestimate the actual effect for a cassava plant producing roots with waxy starch. Perhaps the most relevant study to assess the biological impact of waxy starch in cassava is the ongoing work to evaluate waxy transgenic cassava developed at Wageningen University (Koehorst-van Putten et al., 2012). The Indonesian cultivar *Adira 4* was transformed using the antisense approach to generate versions of the same genotype but with amylose contents ranging from 2 to 10%. The advantage of that study is that basically the same genotype producing contrasting starch types were compared. The comparison of non-transformed versus transformed versions of the cultivar indicated that there was no major difference for FRY. This agrees with results in Thailand and Colombia to develop commercial waxy varieties based on the spontaneous mutation as well as with the results presented in this study. Unfortunately, however, the authors (Koehorst-van Putten et al., 2012) did not report on DMC in that research article.

Results with waxy cassava agree, to some extent, with those reported for maize and other cereals. Waxy maize hybrids had higher moisture at harvest (22.7 versus 21.2%) and lower yields (9.33 versus 9.66 t ha^-1^) that the normal starch counterparts (Fergason, 2001). Similarly, Oscarsson et al. (1998) found that waxy barley varieties were among the lowest yielding and with lowest starch content within a group of 10 varieties. Rooney et al. (2005) reported a 17% reduction in yield in waxy sorghum compared to non-waxy counterparts derived from the same population, while Jampala et al. (2012) found no effect of starch type on grain yield in a sorghum population segregating for the waxy trait. In a study of waxy wheat lines, they were shown to have lower flour yields but the same grain yield as commercial checks (Graybosch et al., 2003).

It is not clear if the low DMC in waxy starch cassava genotypes is related to the biosynthesis of amylopectin in these materials (e.g., pleiotropic effect) or else if the original mutation at the GBSS locus is linked to other loci influencing DMC. If the latter hypothesis is correct, then further sexual crosses among amylose-free cassava genotypes should increase the chances of breaking the eventual linkage between waxy starch and lower-than-desirable DMC. This study provides an unbiased estimate of the biological impact of the waxy starch mutation in cassava without any previous selection: a reduction of less than 1% in DMC. The larger penalty observed in ongoing breeding work (3–4%) may be explained, as suggested above, by the size of populations used for selecting waxy starch versus wild types.

As it is the case waxy maize grains and their derived starch (Elbehri and Paarlberg, 2003), it can be expected that waxy cassava will be sold for a premium price, thereby economically compensating the eventual lower DMC in the roots. The Global Cassava Development Strategy (Food and Agriculture Organization/International Fund for Agriculture Development [FAO/IFAD], 2000) identified the need to strengthen markets for cassava products as a key strategy to realize the impact that this crop can have in the livelihood of millions of people. It is envisioned that developing competitive commercial varieties with amylose-free starch will strengthen the already thriving market for cassava starch, which ultimately helps not only the industry but farmers as well.

## Author Contributions

AK conducted the basic research and took field data as part of her M.Sc dissertation work. She also contributed with data handling and analysis; SS and FC are the assistants of the cassava breeding program and contributed with the original planting of the experiment and continue with further work on the subject; NM is the assistant in charge of making the crosses in the cassava breeding program. All sexual seed used to generate the materials evaluated in this study were produced by his team; DD is an expert in food technology. Many articles describing the functional properties of waxy cassava were co-authored by him; HC is the senior cassava breeder at CIAT and was involved in this project from the very beginning by asking the question that this research attempted to answer. He also contributed with the field design.

## Conflict of Interest Statement

The authors declare that the research was conducted in the absence of any commercial or financial relationships that could be construed as a potential conflict of interest.
